# Successful pregnancy and delivery after ovulation induction therapy in a woman with congenital hypogonadotropic hypogonadism: a case report

**DOI:** 10.1186/s12884-023-05682-7

**Published:** 2023-05-11

**Authors:** Yu Liang, Xiaokui Yang, Ying Li, Lingling Lei, Yonglian Lan, Shuyu Wang

**Affiliations:** Department of Human Reproductive Medicine, Beijing Obstetrics and Gynecology Hospital, Capital Medical University, Beijing Maternal and Child Health Care Hospital, Beijing, 100026 China

**Keywords:** Congenital hypogonadotropic hypogonadism, CHH, Female, Gonadotropin therapy

## Abstract

**Background:**

Congenital hypogonadotropic hypogonadism (CHH) is a rare disorder resulting from a deficient secretion of the episodic gonadotropin-releasing hormone, leading to delayed or absent puberty and infertility. In female patients with CHH, the most commonly used treatment is gonadotropin (Gn) therapy. Due to the rarity of the disease in females, there are limited case reports available. This article offers a management approach for this unusual disease that can be helpful for clinicians.

**Case presentation:**

We report the case of a 29-year-old woman who successfully achieved pregnancy and delivered healthy twin girls after ovulation induction therapy. The patient was diagnosed with CHH at 18 years of age due to primary amenorrhea and the absence of secondary sexual characteristics. After experiencing infertility for three years, the patient sought medical assistance for conceiving. The patient was treated with gonadotropin therapy due to anovulation. In her first treatment cycle, the initial dose of HMG used for treatment was 75IU, which was increased to 150IU after six days. However, the cycle was canceled due to follicular dysplasia. In the second cycle, the treatment began with an initial dose of 150IU, and the follicles grew normally, but the estrogen level was low. Consequently, the treatment was interrupted. In a third ovulation stimulation cycle, HMG was adjusted to 150IU, and recombinant LH was added. After 12 days of ovulation, three mature follicles grew, the estrogen level was normal,and the treatment resulted in successful ovulation and subsequent pregnancy. At 35 weeks of gestation, the patient underwent a cesarean section and delivered two healthy female infants weighing 2,405 g and 2,755 g with an Apgar score of 10/10.

**Conclusions:**

Early diagnosis and timely and appropriate hormone replacement therapy are important for future pregnancy. Ovulation induction therapy is necessary to stimulate fertility. Gn therapy is a feasible and effective treatment for reproduction in CHH females, but the selection of Gn type and dosage must be personalized to maximize fertility outcomes. Effective treatment is available not only for inducing estrogenization and promoting fertility, but also for addressing concerns about psychological and emotional well-being.

## Background

Congenital hypogonadotropic hypogonadism (CHH) is a rare disorder which is clinically characterized by incomplete or absent puberty and infertility due to failed secretion of the episodic gonadotropin-releasing hormone (GnRH), a key neuropeptide that controls mammalian reproduction [1]. The incidence of CHH in males is reported to be around 1/4,000–10,000 [2], with a higher prevalence in males compared females, with a male to female ratio 2:1 to 5:1 [3]. Due to the limited number of female patients affected by CHH there is currently no internationally accepted therapeutic schedule [4].

In women, CHH presents as primary amenorrhea and infertility, which is caused by impaired pituitary secretion of gonadotropins, luteinizing hormone (LH), and follicle-stimulating hormone (FSH), leading to impaired ovarian stimulation [5]. To achieve pregnancy, women with CHH require effective ovulation induction. However, the optimal strategy for inducing fertility in females with CHH is not yet clearly understood.

Gonadotropin (Gn) therapy is the most commonly used treatment in female patients with CHH seeking fertility. The aim of this treatment is to obtain a single ovulation while avoiding multiple pregnancies [4]. However, due to chronic deficiency of gonadotrophins in CHH, the pituitary of females with CHH usually requires several days or weeks to be primed before ovulation induction [6]. Therefore, the total amount of gonadotropin used and the duration of ovarian stimulation were significantly higher than in normal patients [6]. It should be emphasized that the origin of low anti-Mullerian hormone (AMH) levels in CHH is not a true reflection of a patient’s ovarian reserve or fertility odds. AMH is produced by granulosa cells in the recruited primary, secondary, pre-antral, and early antral follicles. Therefore, the administration of FSH in CHH women may increase AMH levels by promoting follicle formation [7]. Successful ovulation induction can be achieved with carefully titrated doses of Gn, which prevent multiple pregnancies and ovarian hyperstimulation. We present a case of an infertile woman affected by CHH and primary amenorrhea who achieved pregnancy and delivery after ovulation induction therapy.

## Case presentation

The patient, a 29-year-old Chinese woman had a medical history of short stature which caused evaluation at the local hospital. At 14 years old, the patient was then treated with growth hormone for one year due to her short stature. Chromosome karyotype analysis using G-banding showed no abnormality (46, XX). At 18 years old, the patient sought medical treatment due to primary amenorrhea and to the absence of secondary sexual characteristics and was diagnosed with hypogonadotropic hypogonadism due to low levels of gonadotropins (< 0.10 mIU/mL) and sex steroids. A GnRH stimulation test was performed. Briefly, 100 µg GnRH was injected intravenously. Prior to administration (0 min) and + 15, +30, + 60 and + 120 min, venous blood was taken to measure serum FSH and LH to examine pituitary and ovarian function. The LH peak value was 10.6 IU/mL, which appeared 60 min after GnRH injection, and the FSH peak value was 9.3 mIU/mL, which appeared 90 min after GnRH injection. These results indicated that a hypothalamic disorder was the cause of the hypogonadotropic hypogonadism. The patient also underwent standardized olfactory test and demonstrated adequate olfactory functioning. Magnetic resonance imaging (MRI) was normal and other hormone levels were standard. After excluding functional GnRH deficiency related to malnutrition, excessive exercise, or stress, the patient was diagnosed with CHH. The patient received estrogen therapy for six months and cyclic estrogen and progesterone treatment to induce secondary sexual characteristics.

At 29 years old, the patient visited the hospital seeking treatment for pregnancy. The patient had been married for three years with a normal sexual life and was 150 cm tall, weighed 45 kg, and had a body mass index (BMI) of 20 kg/m^2^. Physical examination showed Tanner stage 1 breasts and Tanner stage 4 pubic hair. Transvaginal ultrasonography showed the.

uterine size of 3.6*3.6*2.7 cm, the myometrial echo was uniform, and the thickness of the endometrium was 3 mm. Bilaterally, the ovaries were about 18-20 mm in size, and 3–4 small follicles could be dimly seen in each ovary. Endocrine hormonal profile test results showed levels of FSH 0 mIU/mL (reference range, 2.5–10.2 mIU/mL in follicular phase of the menstrual cycle), LH 0 mIU/mL (reference range, 1.9–12.5 mIU/mL in follicular phase of the menstrual cycle), and E2 16.5 pg/mL (reference range, 19.5-144.2 pg/mL in follicular phase of the menstrual cycle), progesterone (P) 0.07 ng/mL (reference range, 0-1.4 ng/mL in follicular phase of the menstrual cycle), total testosterone (T) 15.7 ng/dL (reference range, 8–35 ng/dL), anti-Müllerian hormone (AMH) 4.78 ng/mL (reference range, 0.21–11.45 ng/mL), thyroid-stimulating hormone (TSH) 1.04 µIU/mL (reference range, 0.55–4.78 µIU/mL), free triiodothyronine (FT3) 3.64 pg/mL (reference range, 2.30–4.20 pg/mL), and free thyroxine (FT4) 1.50 ng/dL (reference range, 0.90–1.80 ng/dL). Routine blood tests and biochemical laboratory test results were normal. The results of the analysis of the husband’s semen was also normal.

**Fig. 1:**
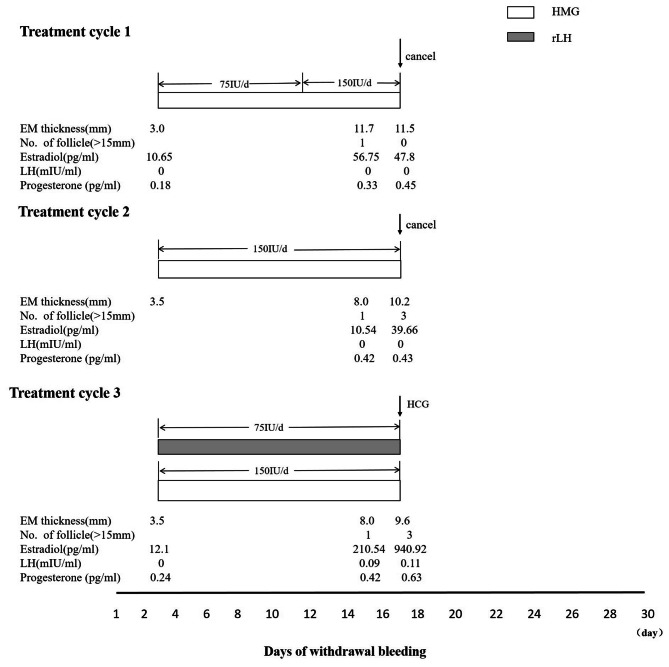
Clinical course of ovarian stimulation treatment cycles. hMG, human menopausal gonadotropin; hCG, human chorionic gonadotropin; EM, endometrium; LH, luteinizing hormone; IU, international unit

To induce ovulation, the patient received three cycles of ovulation induction via gonadotropin therapy using a combination of human menopausal gonadotropin (HMG; Menogon, Ferring GmbH, Kiel, Germany) and recombinant LH (rLH; Luveris; Merck Serono S.p.A., Modugno, Italy) (Fig. 1). Follicle growth was monitored by ultrasound and serum estradiol measurements every 3–4 days.

During the first treatment cycle, ovarian stimulation was initiated on the third day of withdrawal bleeding using a low-dose incremental scheme of 75IU HMG per day. On.

the 9th day of the follicle stimulation, HMG dosage was increased to 150IU per day due to a lack of ovarian response. Unfortunately, on the 15th day of follicle stimulation, follicle atrophy occurred, and the estrogen level decreased, resulting in the termination of the treatment cycle.

In the second treatment cycle, since the patient had not received pituitary hormone stimulation in some time, and a low dose of HMG was insufficient to promote follicle growth, the initial dose of HMG was increased to 150IU per day. On the 14th day of the follicle stimulation, the maximum follicle diameter was 19.8 mm, but estrogen levels remained low, at 39.66pg/mL (compared to 200–300 pg/mL of estrogen in a normal mature follicle). Consequently, the treatment was interrupted.

Clinicians considered that the final maturation of follicles might be difficult due to the lack of LH, and so recombinant LH was added to the third treatment cycle. Ovulation stimulation employed HMG 150IU per day along with rLH. On the 15th day of the follicle stimulation, three mature follicles, each over 15 mm in diameter, were observed, and estrogen levels were 940.92 pg/mL. The patient was given a subcutaneous dose of 250 µg of human chorionic gonadotropin (HCG, Ovidrel, Merck Serono S.p.A.) to stimulate ovulation, and sexual intercourse was recommended the following day.

After ovulation, the luteal phase was supported by administration of 800 mg of micronized progesterone (Utrogestan, Laboratoires Besins International, Paris, France) per day. Eleven days after ovulation, serum HCG test levels were 60.56 mIU/mL. At six weeks and three days of gestation, abdominal ultrasonography revealed a dual gestational sac within the uterus and both fetal heartbeats could be detected. The patient developed uterine contractions and vaginal bleeding beginning at 35 weeks of gestation. Ultrasound examination demonstrated that the first fetus had a breech presentation, while the second fetus had a head presentation. The obstetrician opted for implementation of a cesarean section. The patient delivered two healthy female infants weighing 2,405 g and 2,755 g with an Apgar score of 10/10.

## Discussion

CHH exhibits significant genetic heterogeneity, with only about 30% of cases having a familial link, while the vast majority are sporadic [8]. The epidemiological difference in prevalence between males and females remains unclear. CHH has various inheritance patterns, such as autosomal recessive and dominant, and X-linked recessive [8]. Rare ANOS1 variants, located on the X chromosome, account for around 10% of cases and are among the most prevalent genetic causes [9]. X-linked inheritance may explain the lower prevalence of CHH in females. Due to the difficulty in distinguishing CHH from other GnRH deficiencies, partial CHH in females may be underdiagnosed [4]. Here, we present the case of a woman with CHH who achieved a successful pregnancy and delivery following ovulation induction therapy.

Pulsatile secretion of GnRH is crucial for the development of secondary sexual characteristics and reproductive functioning in both sexes [10]. GnRH-producing neurons differ from other neuroendocrine cells in that they originate in the olfactory placode, migrate extensively during embryonic development, and finally settle in the hypothalamus region at birth [7]. As maternal hCG primarily drives the development of external genitalia, there are usually no abnormalities in the development of the internal and external genitalia in CHH females [11]. In addition, childhood can be considered a hypogonadal state due to the relative quiescence of the GnRH pulse generator, making early diagnosis challenging [4].

Puberty initiation is dependent on the activation of the hypothalamic-pituitary-gonadal (HPG) axis. The HPG axis is gradually reactivated, leading to an increase in GnRH secretion. Pulsatile GnRH release stimulates the synthesis and release of gonadotropins such as FSH and LH by pituitary. These hormones, in turn, stimulate the gonads to produce sex steroids and gametes [12]. Elevated estrogen levels promote uterine growth, breast development, and the onset of the menstrual cycle. GnRH deficiency results in incomplete or absent puberty development, with CHH females exhibiting varying degrees of breast underdevelopment, with over 90% of them experiencing primary amenorrhea [13]. Patients with no breast development or menstruation over the age of 15 are advised to undergo a thorough examination to obtain an accurate diagnosis [8]. Timely and appropriate hormonal replacement therapy (HRT) is critical for CHH female patients. HRT can induce puberty, maintain secondary sex characteristics, stimulate growth in stature, achieve peak bone mass, and promote patients’ psychosexual development. The patient in this report was diagnosed at 18 years old and received hormone replacement therapy. However, her pubertal development was suboptimal. Delaying estrogen replacement and administering an insufficient estrogen dose may have caused breast dysplasia and short stature. As a result, HRT must be handled with great care, and where feasible, such treatment should be supervised by a specialist since it must be customized for fertility treatment.

Inducing gonadotropins via GnRH is essential to produce gametes and achieving fertility. Based on the two-cell–two-gonadotropin theory [14], the synergistic effect of FSH and LH is critical for the normal development of follicles. FSH promotes the recruitment of secondary ovarian follicles in the ovarian antrum, regulates the development and atresia of follicles, while LH stimulates theca cells to produce androgens which are converted to estrogens in granulosa cells by FSH-induced aromatase [15]. LH is also necessary for optimal follicular development and ovulation. Due to GnRH deficiency, CHH females present impairment in follicular terminal growth and maturation. Therefore, ovulation induction treatment is required for these women to be able to conceive. Women with CHH require simultaneous ovarian and uterine priming due to chronic gonadotrophin deficiency. Ovarian priming is achieved with HMG or recombinant FSH and LH, while uterine priming is achieved with estrogen [6].

Ovulation can be achieved by pulsatile GnRH or gonadotropin stimulation. Pulsatile GnRH hormone pumping can restore the physiological secretion of pituitary gonadotropin, resulting in ovulation [16]. The pump is controlled by a microcomputer, with the dose and frequency of GnRH release able to be adjusted precisely [17]. The main advantage of this treatment is lower rates of multiples and ovarian hyperstimulation syndrome, but as it needs a special pump and continuous subcutaneous injections, it is not widely used. Gonadotropin stimulation followed by HCG to trigger ovulation is a more commonly used treatment option for inducing fertility in CHH patients. Gn is currently available in various formulas and usage methods and is commonly used in ovulation induction therapy.

In this report, the initial dose of HMG used for treatment was 75IU, which was increased to 150IU after six days. However, the cycle was canceled due to follicular dysplasia. In the second cycle, the treatment began with an initial dose of 150IU, and the follicles grew normally, but the estrogen level was low. An IVF treatment on an HH woman which confirmed that LH-free stimulation induces normal follicular growth accompanied by low E2 production and without normal oocyte maturation and fertilization ability [18]. The direct cause of poor oocyte quality was found to be insufficient LH secretion [18]. HMG contains LH and FSH in a ratio of 1:1, and typically, a daily HMG dose of 75 to 150 IU is sufficient to induce ovulation in CHH females. However, in this report, the patient had experienced multiple failed ovulation inductions with HMG doses ranging between 75 and 150 IU/d, suggesting that patients may require a higher LH threshold to achieve full maturation and ovulation of oocytes. In the third and final cycle of ovulation stimulation, recombinant LH was added based on the results of the previous treatment. After 12 days of ovulation, three mature follicles grew, and the estrogen levels remained normal. Follicular growth was significantly enhanced through LH supplementation, consistent with previous results [19, 20]. Studies with HH women have shown that the minimum LH level necessary for normal follicular growth in HH patients is debated, and personalized treatment with an optimal LH/FSH ratio is required according to the ovarian response [21, 22].

However, it is worth noting that in this report, HMG should have returned to a lower dose after supplementation with r-LH. In retrospect, a lower dose would have been more prudent, and perhaps the patient would not have eventually developed three mature follicles, which can also prevent multiple pregnancies. In a young woman in her very first opportunity to conceive, triggering 3 mature follicles may have been considered risky. It is also important to note that the patient had experienced multiple failed ovulation inductions (including several times at other hospital), and had a strong desire to attempt pregnancy. After fully explaining the possible risks, including multiple pregnancies and hyperstimulation syndrome, the patient chose to attempt pregnancy. While healthy twins ultimately resulted, this outcome could have been much more complicated. This report serves as a valuable lesson for clinicians to carefully consider risks and benefits of each treatment option for their patients.

CHH is a genetically heterogeneous disorder, with more than 30 gene mutations identified to date [23]. Oligogenicity has also been reported in CHH [23]. Patients with a positive family history should consider genetic counseling. In this report, the patient declined genetic testing, but we recommended genetic testing to determine the genetic origin of the disorder, enabling early assessment of offspring to ensure early diagnosis.

When dealing with women with CHH, a holistic approach is important, addressing not only fertility but also body image, psychosexual development, and relationship confidence [6]. Psychological and peer support should be strongly considered to address these issues [3, 24].

Overall, CHH is rare in females and it can be difficult to diagnose. Clinicians should pay attention to constitutional delays in growth and puberty, as early diagnosis and timely and appropriate hormone replacement therapy are important to increase uterine size and to promote psychosexual development which are ultimately important for future pregnancy. Ovulation induction therapy is necessary to stimulate fertility. Gn therapy is a feasible and effective treatment for reproduction in CHH females, but the selection of Gn type and dosage must be personalized to maximize fertility outcomes. Effective treatment is available not only for inducing estrogenization and promoting fertility, but also for addressing concerns about psychological and emotional well-being.

## Data Availability

All data generated or analyzed during this study are included in this published article.

## Abbreviations

CHH: Congenital Hypogonadotropic hypogonadism
Gn: Gonadotropin
HMG: Human Menopausal Gonadotropin
GnRH: Gonadotropin-Releasing Hormone
LH: Luteinizing Hormone
FSH: Follicle-Stimulating Hormone
MRI: Magnetic Resonance Imaging
BMI: Body Mass Index
P: Progesterone
T: Testosterone
AMH: Anti-Müllerian Hormone
TSH: Thyroid-Stimulating Hormone
FT_3_: Free Triiodothyronine
FT4: Free Thyroxine
rLH: recombinant LH
HCG: Human Chorionic Gonadotropin
HPG: Hypothalamic-Pituitary-Gonadal
